# High-efficiency X-band electromagnetic wave absorption in Co–Al engineered Ba–Sr hexaferrites: role of cationic substitution and microstructural tuning

**DOI:** 10.1039/d5ra08548a

**Published:** 2025-12-15

**Authors:** Pallavi S. Salunke, Manisha R. Patil, Kanak S. Alone, Akash V. Fulari, Vinod K. Barote, Maheshkumar L. Mane, R. H. Kadam, Suresh T. Alone, Sagar E. Shirsath, Vinod N. Dhage

**Affiliations:** a Advanced Materials and Nanotechnology Research Laboratory, Department of Physics, MES Abasaheb Garware College Pune 411004 MS India vn_dhage@rediffmail.com; b Department of Physics, Deogiri College Chhatrapati Sambhajinagar 431001 MS India; c School of Engineering and Science, SRM University Amaravati 522502 AP India; d Symbiosis Centre for Research and Innovation, Symbiosis International (Deemed University) Pune 412115 MS India; e Department of Physics, Sant Dnyaneshwar Mahavidyalaya Soegaon 431120 Maharashtra India; f Shikshan Maharshi Guruvarya R. G. Shinde Mahavidyalaya Paranda Dharashiv 413502 MS India; g Physics Department, Shrikrishna College Gunjoti Dharashiv 413606 MS India; h Department of Physics, Rajarshi Shahu College Pathri Chhatrapati Sambhajinagar 431111 MS India; i Physics Department, Vivekanand College Chhatrapati Sambhajinagar 431005 MS India; j School of Materials Science and Engineering, University of New South Wales Sydney NSW 2052 Australia s.shirsath@unsw.edu.au shirsathsagar@hotmail.com

## Abstract

The growing demand for high-performance electromagnetic (EM) absorbers and interference (EMI) shielding materials has motivated the design of structurally tunable ferrites with balanced dielectric and magnetic losses. In this study, crystalline Ba_0.5_Sr_0.5_Y_1.0_Fe_11−*x*_(Co_*x*/2_Al_*x*/2_)O_19_ (*x* = 0.00–1.00) hexaferrites were synthesized *via* a sol–gel auto-combustion route to investigate the correlation between multi-cation substitution, microstructure, and microwave absorption. Rietveld-refined X-ray diffraction confirmed single-phase M-type hexagonal symmetry (*P*6_3_/*mmc*) with systematic lattice contraction from 693.53 Å^3^ (*x* = 0.0) to 671.27 Å^3^ (*x* = 1.0) due to smaller Co^2+^ and Al^3+^ ionic radii. Field emission scanning electron microscopy and energy-dispersive X-ray analyses revealed dense, homogeneous microstructures with uniform dopant distribution. Magnetic measurements showed a progressive softening with substitution saturation magnetization (*M*_s_) decreased from 54.79 emu g^−1^ (*x* = 0.0) to 41.80 emu g^−1^ (*x* = 1.0), while coercivity (*H*_c_) dropped from 1788.8 Oe to 77.7 Oe attributed to dilution of Fe^3+^–O–Fe^3+^ superexchange and reduced anisotropy constant (*K*_1_ ≈ 10^5^ to 10^3^ erg cm^−3^). The optimized composition, *x* = 0.50, achieved outstanding microwave absorption with a minimum reflection loss (RL_min_) of −47.58 dB at 9.27 GHz (6 mm thickness) and an effective absorption bandwidth of 0.609 GHz, confirming nearly complete EM attenuation. Dielectric and magnetic spectra revealed that balanced permittivity (*ε*′, *ε*″) and permeability (*µ*′, *µ*″) ensured impedance matching (*Z*_in_ ≈ *Z*_0_), while enhanced attenuation constant (*α*) and reduced eddy current loss promoted multi-mechanistic energy dissipation. These findings demonstrate that Co–Al co-substitution effectively tailors lattice strain, anisotropy, and interfacial polarization, providing a rational design strategy for high-efficiency microwave absorbers and EMI-shielding hexaferrites in the X-band region.

## Abstract 2

The growing demand for high-performance electromagnetic (EM) absorbers and interference (EMI) shielding materials has motivated the design of structurally tunable ferrites with balanced dielectric and magnetic losses.

## Graphical abstract

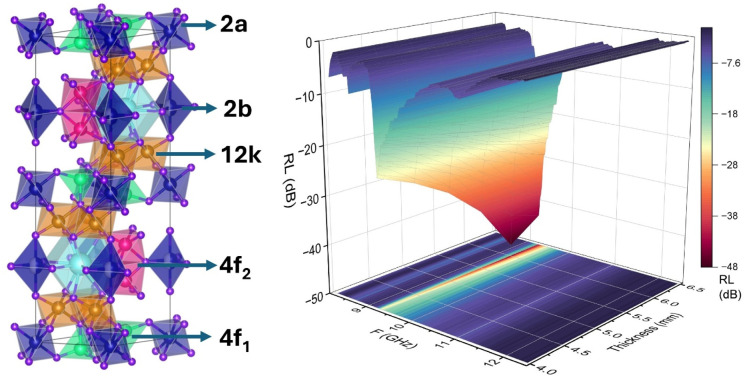

## 1. Introduction

The rapid expansion of wireless communication, radar, and high-speed electronics has intensified electromagnetic (EM) pollution, creating an urgent need for high-efficiency microwave absorbers and EMI-shielding materials to ensure signal integrity and stealth performance.^1–6^ Recent advances in high-performance microwave absorbers highlight that achieving broad, tunable, and efficient attenuation requires deliberate control of interfacial polarization, charge–spin interactions, and structure-dependent anisotropy, rather than relying solely on bulk magnetic or dielectric loss.^7–10^ Hollow-engineered architectures, as demonstrated by Xiao *et al.*,^11^ create internal cavities and multi-scale interfaces that strengthen interfacial polarization, enhance impedance matching, and promote repeated EM-wave scattering for bandwidth expansion. High-entropy interface engineering induces electronic redistribution and lattice distortion, creating multiple polarization pathways that markedly enhance dielectric loss and broadband absorption.^12–14^ Likewise, studies on advanced composite absorbers show that heterointerfaces and anisotropy modulation are vital for overcoming the intrinsic frequency selectivity and narrowband limitations of single-phase materials.^15^

Among the various candidate materials, hexagonal ferrites (hexaferrites) stand out due to their unique capability to attenuate EM waves through both dielectric and magnetic loss mechanisms.^16–18^ Their high electrical resistivity suppresses eddy current losses, while their intrinsic ferrimagnetic order allows for tunable magnetic permeability two essential features for impedance matching.^19,20^ In particular, the M-type hexaferrites, with a general formula AFe_12_O_19_ (where A = Ba^2+^ or Sr^2+^), exhibit high Curie temperatures, large magnetocrystalline anisotropy fields, chemical stability, and are cost-effective to synthesize. These properties make them particularly attractive for room-temperature microwave and EMI shielding applications. Structurally, M-type ferrites crystallize in a magnetoplumbite-type hexagonal lattice (space group: *P*6_3_/*mmc*) composed of alternating S (spinel) and R (hexagonal) blocks, arranged in an RSRS stacking sequence. The magnetic behavior arises from Fe^3+^ ions distributed over five crystallographic sites three spin-up (12k, 2a, 2b) and two spin-down (4f_1_, 4f_2_) whose net alignment dictates the overall magnetization and anisotropy.^20,21^

However, despite these advantages, pure M-type ferrites suffer from limitations in bandwidth and absorption intensity due to fixed ferromagnetic resonance (FMR) frequencies and rigid magnetic anisotropy.^22,23^ To improve the absorption bandwidth and achieve better impedance matching with free space, modifications are necessary to tailor both permittivity (*ε* = *ε*′ − *iε*″) and permeability (*µ* = *µ*′ − *iµ*″).^24,25^ This is typically accomplished through cationic substitution, which alters spin alignment, carrier mobility, and the magnetic anisotropy field (*H*_a_).^26,27^ A key objective in materials engineering for microwave absorbers is to balance dielectric and magnetic losses (tan *δε* = *ε*″/*ε*′ and tan *δµ* = *µ*″/*µ*′) while simultaneously tuning the attenuation constant and achieving *Z*_in_ ≈ *Z*_0_ for maximal absorption.

Among cationic substitution strategies, dual or multi-site doping has emerged as a particularly powerful route. For instance, substituting Fe^3+^ with a divalent-trivalent cation pair, such as Co^2+^ and Al^3+^, enables charge neutrality while synergistically adjusting both magnetic and dielectric properties.^21,28^ Co^2+^, being a magnetic ion with high spin–orbit coupling, enhances uniaxial anisotropy and shifts the FMR toward the GHz regime, boosting magnetic loss. On the other hand, Al^3+^, with its smaller ionic radius and non-magnetic nature, increases electrical resistivity and modulates dielectric polarization by affecting grain boundaries and oxygen vacancy concentrations.^29–31^ Simultaneously, rare-earth substitution with Y^3+^ has been shown to introduce lattice distortion and modify sublattice interactions, thereby influencing spin dynamics and interfacial polarization.^32–34^

While many prior reports have explored the individual effects of Co^2+^ or Al^3+^ doping in ferrites, relatively few have investigated their combined substitution, especially in conjunction with rare-earth elements in Y-type or Ba–Sr mixed systems. Furthermore, comprehensive studies that correlate crystallographic structure, lattice strain, magnetic hysteresis, complex permittivity/permeability, and EMI shielding metrics remain limited.

In this work, we systematically investigate the multi-cation co-substitution strategy in Ba_0.5_Sr_0.5_Y_1.0_Fe_11−*x*_(Co_*x*/2_Al_*x*/2_)O_19_ particles (*x* = 0.00, 0.25, 0.50, 0.75, and 1.00), synthesized *via* the sol–gel auto-combustion method to achieve fine particle size and high phase purity. The structural evolution is probed through Rietveld-refined X-ray diffraction, while morphological features are examined *via* field emission scanning electron microscopy (FESEM) and elemental mapping. Magnetic behavior is studied using hysteresis measurements and complex magnetic parameters, while microwave and shielding properties are characterized in the X-band (8–12.5 GHz) through analysis of complex permittivity, permeability, reflection loss, attenuation constant, eddy current behavior, and shielding effectiveness (SEA, SER, SET). Special emphasis is placed on impedance matching, magnetic resonance, and dielectric relaxation mechanisms, in light of the Maxwell–Wagner polarization model and Koops' theory.

Our results reveal that compositions with *x* = 0.50 and 0.75 offer an optimal trade-off between magnetic and dielectric losses, achieving enhanced microwave absorption with wide effective absorption bandwidths (EABs), low return loss, and superior shielding efficiency. These findings not only underscore the potential of Co–Al co-substitution in tailoring multifunctional properties of hexaferrites but also provide mechanistic insight for the rational design of high-performance absorbers for electromagnetic stealth and EMI suppression.

## 2. Methods and materials

Analytical-grade precursors with ≥99.9% purity (Sigma-Aldrich) were used for the synthesis. The starting reagents included strontium nitrate (SrN_2_O_6_), barium nitrate (BrN_2_O_6_), cobalt nitrate (Cr(NO_3_)_2_·6H_2_O), aluminum nitrate (Al(NO_3_)_3_·9H_2_O), yttrium(iii) nitrate hexahydrate [Y(NO_3_)_3_·6H_2_O], ferric nitrate (FeN_3_O_9_·9H_2_O), and citric acid (C_6_H_8_O_7_·H_2_O) as a complexing/fueling agent. Crystalline Ba_0.5_Sr_0.5_Y_1.0_Fe_11−*x*_Co_*x*/2_Al_*x*/2_O_19_ (*x* = 0.00, 0.25, 0.50, 0.75, 1.00) powders were prepared through the sol–gel auto-combustion route.^35^

Stoichiometric quantities of the respective metal nitrates were dissolved in approximately 200 mL of deionized water under constant stirring to obtain a transparent homogeneous solution. Citric acid was added as a chelating agent to promote uniform cation distribution. The pH of the mixed solution was carefully adjusted to around 8 by the slow addition of aqueous ammonia, facilitating gel formation. Continuous heating and stirring caused the solution to transform into a viscous gel, which subsequently underwent a spontaneous exothermic combustion reaction, yielding a voluminous and fluffy precursor powder. The as-burnt powders were calcined at 1200 °C for 6 h in a muffle furnace to achieve phase-pure, crystalline M-type hexaferrites with improved thermal stability.

Phase identification and structural analysis were carried out using X-ray diffraction (XRD, Rigaku Ultima IV, Cu Kα = 1.5406 Å) within a 2*θ* range of 20°–80°. Rietveld refinement of the XRD data was performed using the FullProf Suite to extract accurate lattice parameters and confirm single-phase formation. Surface morphology was examined with field-emission scanning electron microscopy (FESEM, Carl Zeiss EVO 50), and the average grain size was determined using the linear-intercept method in ImageJ software. Elemental composition and distribution uniformity were verified through energy-dispersive X-ray spectroscopy (EDAX). Magnetic hysteresis loops were recorded at room temperature using a Lakeshore 7410 vibrating sample magnetometer (VSM) under an applied magnetic field of ±18 kOe.

For microwave absorption studies, each ferrite powder was blended with epoxy resin in an 80 : 20 wt% ratio to form a uniform composite paste. The mixture was pressed into rectangular pellets using a hydraulic press to ensure consistent geometry for X-band testing. Reflection loss and electromagnetic parameters were measured in the 8–12 GHz range using a Keysight ENA E5063A vector network analyzer. These measurements provided quantitative evaluation of the electromagnetic attenuation and absorption efficiency of the prepared ferrite particles.

## 3. Results and discussion

### 3.1 Structural properties

**Fig. 1:**
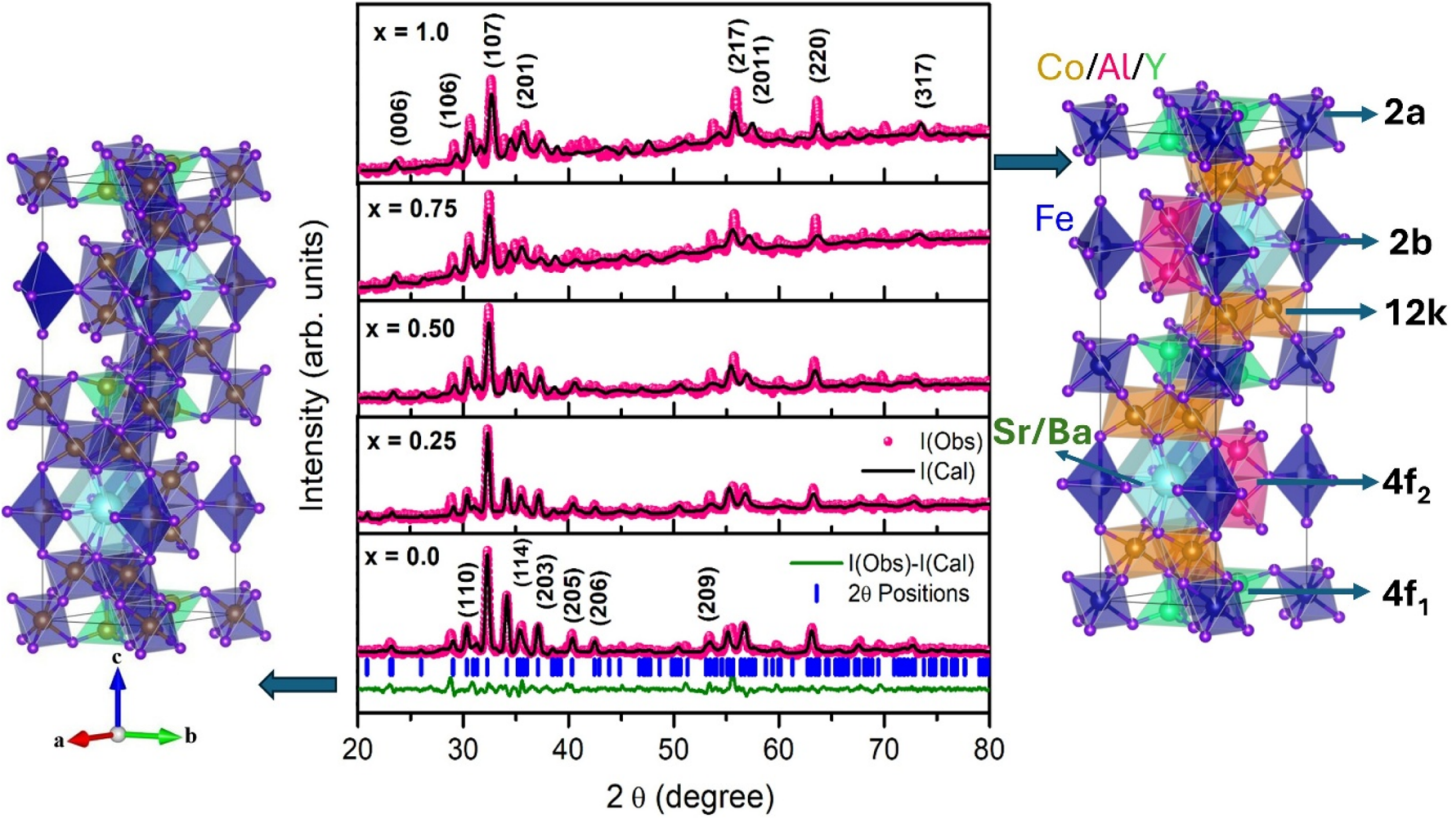
(Left) Crystal structure of pure Ba_0.5_Sr_0.5_Y_1.0_Fe_11_O_19_ (*x* = 0.0) hexaferrite, illustrating the magnetoplumbite-type structure composed of R and S blocks with Fe^3+^ occupying various crystallographic sites. (Center) Rietveld refinement X-ray diffraction (XRD) patterns of Ba_0.5_Sr_0.5_Y_1.0_Fe_11−*x*_Co_*x*/2_Al_*x*/2_O_19_. (Right) Crystal structure of Co–Al-substituted hexaferrite, with site indexing showing Fe^3+^ sublattice positions. The 2a site remains fully occupied by Fe^3+^ across all compositions, whereas Co^2+^ preferentially substitutes Fe^3+^ at the 12k site and Al^3+^ occupies the 4f_1_ and 4f_2_ sites.

The phase composition and crystallographic structure of the synthesized Ba_0.5_Sr_0.5_Y_1.0_Fe_11−*x*_(Co_*x*/2_Al_*x*/2_)O_19_ (*x* = 0.00–1.00) hexaferrite system were investigated through X-ray diffraction (XRD), and detailed structural information was extracted *via* Rietveld refinement using the FullProf Suite. The Rietveld refined XRD patterns, shown in Fig. 1, exhibit well-resolved and sharp peaks characteristic of the M-type hexaferrite phase with hexagonal symmetry (space group *P*6_3_/*mmc*), affirming the successful formation of a single-phase structure across all compositions.^26,36,37^ No extraneous peaks corresponding to secondary phases or impurity oxides were observed, confirming the phase purity of the samples.

The refined lattice parameters (Table 1) reveal a systematic contraction of the unit cell with increasing Co–Al substitution. Lattice constant was calculated using the formula:1
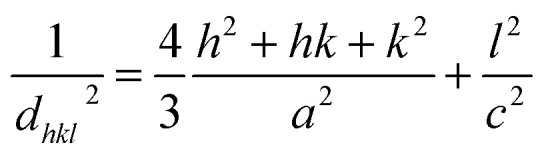


**Table 1:** Rietveld refinement parameters of Ba_0.5_Sr_0.5_Y_1.0_Fe_11−*x*_Co_*x*/2_Al_*x*/2_O_19_

‘*x*’	‘*a*’ (Å)	‘*c*’ (Å)	*c*/*a* ratio	*χ* ^2^	*R*_P_	*R*_WP_	*R*_EXP_
0.0	5.8879	23.1007	3.923	2.47	44.4	27.1	17.23
0.25	5.8746	23.0554	3.925	2.12	54.8	33.1	22.72
0.50	5.8608	23.0088	3.926	3.48	69.2	42.6	22.85
0.75	5.8419	22.9136	3.922	3.66	87.7	56.5	29.57
1.0	5.8335	22.7784	3.905	4.45	83.0	53.7	25.47

Specifically, the lattice constants ‘*a*’ and ‘*c*’ decrease from 5.8879 Å and 23.1007 Å for *x* = 0.00 to 5.8335 Å and 22.7784 Å for *x* = 1.00, respectively. This contraction is attributed to the replacement of Fe^3+^ ions (ionic radius ∼0.645 Å in octahedral coordination) with Al^3+^ (∼0.535 Å) and Co^2+^ (∼0.72 Å) ions. The observed reduction in unit cell volume is listed in Table 1, is in agreement with Vegard's law and further confirms that the substitution occurs homogeneously within the crystal lattice. Interestingly, the *c*/*a* ratio remains relatively stable around ∼3.92–3.93 across the entire substitution series, suggesting that the hexagonal symmetry is preserved despite the lattice contraction.

The goodness of fit in Rietveld refinement, as reflected by *R*_p_, *R*_wp_, and *R*_exp_ values (Table 1), remains within acceptable limits for all compositions. While the reduction in lattice parameters reflects successful substitution, the quality indicators from the Rietveld refinement specifically *R*_p_, *R*_wp_, and *R*_exp_ show a slight increase with *x*, particularly beyond *x* = 0.50. For example, *R*_wp_ increases from 27.1 (*x* = 0.0) to 53.7 (*x* = 1.0), and *R*_exp_ rises from 17.23 to 25.47 across the same range. This may indicate the onset of subtle structural distortions or increasing microstrain due to lattice mismatch as substitution progresses. The increased *R*_p_ and *R*_wp_ values, however, remain within acceptable refinement thresholds and do not suggest any phase decomposition or amorphization.

The Rietveld refinement confirms that Co–Al substitution for Fe in Ba_0.5_Sr_0.5_Y_1.0_Fe_11_O_19_ successfully modifies the lattice while retaining the characteristic M-type hexagonal structure. The observed lattice contraction with increasing *x* is a direct result of ionic size effects and provides an essential foundation for understanding the subsequent changes in magnetic and electromagnetic properties discussed in later sections.

The cation distribution of Ba_0.5_Sr_0.5_Y_1.0_Fe_11−*x*_(Co_*x*/2_Al_*x*/2_)O_19_ was assigned based on the crystallographic site multiplicities and the well-established site preferences of Fe^3+^, Co^2+^, and Al^3+^ in the M-type hexaferrite structure (*P*6_3_/*mmc*). The Fe^3+^ ions occupy five distinct sites, namely 12k, 4f_2_, 4f_1_, 2a, and 2b. Co^2+^ ions preferentially substitute Fe^3+^ at the 12k octahedral site, whereas Al^3+^ ions preferentially occupy the 4f_1_ and 4f_2_ sites, which have lower crystal-field energies. The 2a and 2b sites remain fully occupied by Fe^3+^ at all compositions. The detailed site occupancy values for each dopant level (*x* = 0.00–1.00) are summarized in Table 2.

**Table 2:** Cation distribution, Wyckoff positions, coordination geometry, spin orientation, and site occupancy of all ions in Ba_0.5_Sr_0.5_Y_1.0_Fe_11−*x*_Co_*x*/2_Al_*x*/2_O_19_ (*x* = 0.00–1.00)

Site	Wyckoff position	Coordination	Spin	*x* = 0.00	*x* = 0.25	*x* = 0.50	*x* = 0.75	*x* = 1.00
12k	12k	Octahedral (O_h_)	↑	Fe_6.0_	Fe_5.875_Co_0.125_	Fe_5.75_Co_0.25_	Fe_5.625_Co_0.375_	Fe_5.50_Co_0.50_
4f_2_	4f_2_	Octahedral (O_h_)	↓	Fe_2.0_	Fe_1.9375_Al_0.0625_	Fe_1.875_Al_0.125_	Fe_1.8125_Al_0.1875_	Fe_1.75_Al_0.25_
4f_1_	4f_1_	Tetrahedral (T_d_)	↓	Fe_2.0_	Fe_1.9375_Al_0.0625_	Fe_1.875_Al_0.125_	Fe_1.8125_Al_0.1875_	Fe_1.75_Al_0.25_
2a	2a	Octahedral (O_h_)	↑	Fe_1.0_	Fe_1.0_	Fe_1.0_	Fe_1.0_	Fe_1.0_
2b	2b	Bipyramidal	↑	Fe_1.0_	Fe_1.0_	Fe_1.0_	Fe_1.0_	Fe_1.0_
2d	2d	12-Coordination	—	Ba_0.5_Sr_0.5_	Ba_0.5_Sr_0.5_	Ba_0.5_Sr_0.5_	Ba_0.5_Sr_0.5_	Ba_0.5_Sr_0.5_
2c	2c	8-Coordination	—	Y_1.0_	Y_1.0_	Y_1.0_	Y_1.0_	Y_1.0_
O sites	4e, 6h, 12k	—	—	O_19_	O_19_	O_19_	O_19_	O_19_

The influence of Co–Al substitution on microstructural evolution and densification behavior in Ba_0.5_Sr_0.5_Y_1.0_Fe_11−*x*_(Co_*x*/2_Al_*x*/2_)O_19_ (*x* = 0.00–1.00) was further elucidated through the evaluation of structural parameters listed in Table 3. Unit cell volume was calculated using the relation:2
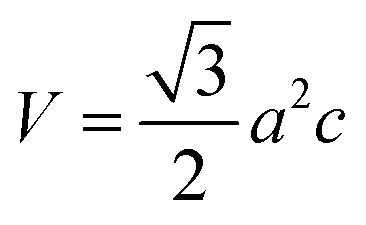


**Table 3:** Cell volume (*V*), X-ray density (*d*_*x*_), bulk density (*d*_B_), percentage porosity (*P*), specific surface area (*S*), crystallite size (*t*) and lattice strain (*ε*) for Ba_0.5_Sr_0.5_Y_1.0_Fe_11−*x*_Co_*x*/2_Al_*x*/2_O_19_

‘*x*’	*V* (Å^3^)	‘*d*_*x*_’ (g cm^−3^)	‘*d*_B_’ (g/cm^3^)	*P* (%)	*S* (m^2^ g^−1^)	‘*t*’ (nm)		*ε* × 10^−4^
						Scherrer	W–H	
0.0	693.53	5.332	4.148	28.55	100	**15.58**	**17.75**	6.75
0.25	689.04	5.351	4.267	25.40	93	**16.29**	**18.14**	8.74
0.50	684.42	5.371	4.378	22.68	88	**16.84**	**19.50**	10.8
0.75	677.20	5.413	4.506	20.13	82	**17.44**	**20.96**	12.3
1.0	671.27	5.445	4.660	16.83	77	**17.97**	**22.10**	13.1

A progressive decrease in unit cell volume (*V*) is observed with increasing substitution content, from 693.53 Å^3^ at *x* = 0.00 to 671.27 Å^3^ at *x* = 1.00. This volume contraction is attributed to the successful replacement of Fe^3+^ ions (ionic radius ∼0.645 Å) with Al^3+^ (∼0.535 Å) and Co^2+^ (∼0.72 Å) ions. The incorporation of these smaller cations into the Fe sublattice introduces compressive lattice strain and leads to overall densification of the crystal structure, without disrupting the fundamental hexagonal symmetry.^38^

The X-ray density (*d*_*x*_), calculated based on the molecular weight and refined lattice parameters, shows a steady increase with substitution, rising from 5.332 g cm^−3^ at *x* = 0.00 to 5.445 g cm^−3^ at *x* = 1.00. This increase reflects the atomic weight contribution of cobalt and aluminum and the reduction in cell volume, which collectively enhance the mass-to-volume ratio. Complementarily, the experimental bulk density (*d*_B_) exhibits a similar upward trend, increasing from 4.148 g cm^−3^ to 4.660 g cm^−3^. The enhancement in bulk density suggests improved particle packing and reduced void fraction within the pelletized samples, likely a result of grain growth and particle agglomeration facilitated by substitution.

The percentage porosity (*P*), derived from the relation:3
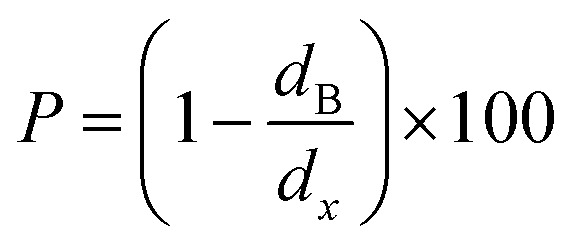
shows a noticeable decline with increasing *x*, decreasing from 28.55% at *x* = 0.00 to 16.83% at *x* = 1.00. This substantial reduction in porosity confirms that Co–Al substitution contributes to densification during sintering, possibly by enhancing atomic diffusion and facilitating grain boundary elimination. The densified structure is expected to reduce the prevalence of non-magnetic grain boundaries, thereby influencing magnetic coupling and dielectric homogeneity in the system.

Crystallite size was evaluated using both the Debye–Scherrer equation and the Williamson–Hall (W–H) method,^39^ which jointly account for size-induced and strain-induced peak broadening in the XRD patterns. A gradual increase in average crystallite size is observed, rising from 15.58 nm at *x* = 0.00 to 17.97 nm at *x* = 1.00 (Scherrer values) (Table 3). The W–H-derived values also follow a similar trend, from 17.75 nm to 22.10 nm across the same substitution range, indicating that substitution promotes crystal growth and reduces lattice imperfections to some extent.

Specific surface area (*S*), calculated from the crystallite size and density, 
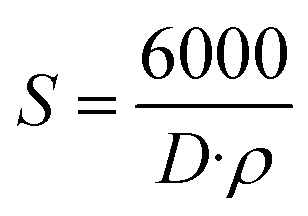
, shows a decreasing trend from 100 m^2^ g^−1^ to 77 m^2^ g^−1^ with increasing *x*. This reduction is coherent with the crystallite growth observed and indicates a slight reduction in surface reactivity and interfacial area. The decline in *S* may influence surface-related phenomena such as microwave attenuation or domain wall pinning, which will be discussed in subsequent sections.

The incorporation of Co^2+^ and Al^3+^ into Fe^3+^ crystallographic sites modifies the structure through multiple interrelated factors. The primary driving force is the ionic radius mismatch, as both Co^2+^ and Al^3+^ ions are smaller than Fe^3+^, leading to lattice shrinkage and increased internal stress. Furthermore, while Fe^3+^ is trivalent, Co^2+^ introduces a divalent charge state, and Al^3+^ is trivalent; thus, the coupled substitution of one Co^2+^ and one Al^3+^ in place of two Fe^3+^ ions maintains overall charge neutrality but alters the local crystal field environment. Co^2+^, particularly in high-spin (d^7^) configuration, tends to introduce additional crystal field anisotropy and can induce Jahn–Teller-like distortions. In contrast, Al^3+^, being non-magnetic with a closed-shell configuration (d^0^), contributes to structural stabilization and local symmetry breaking without adding magnetic interactions.

**Fig. 2:**
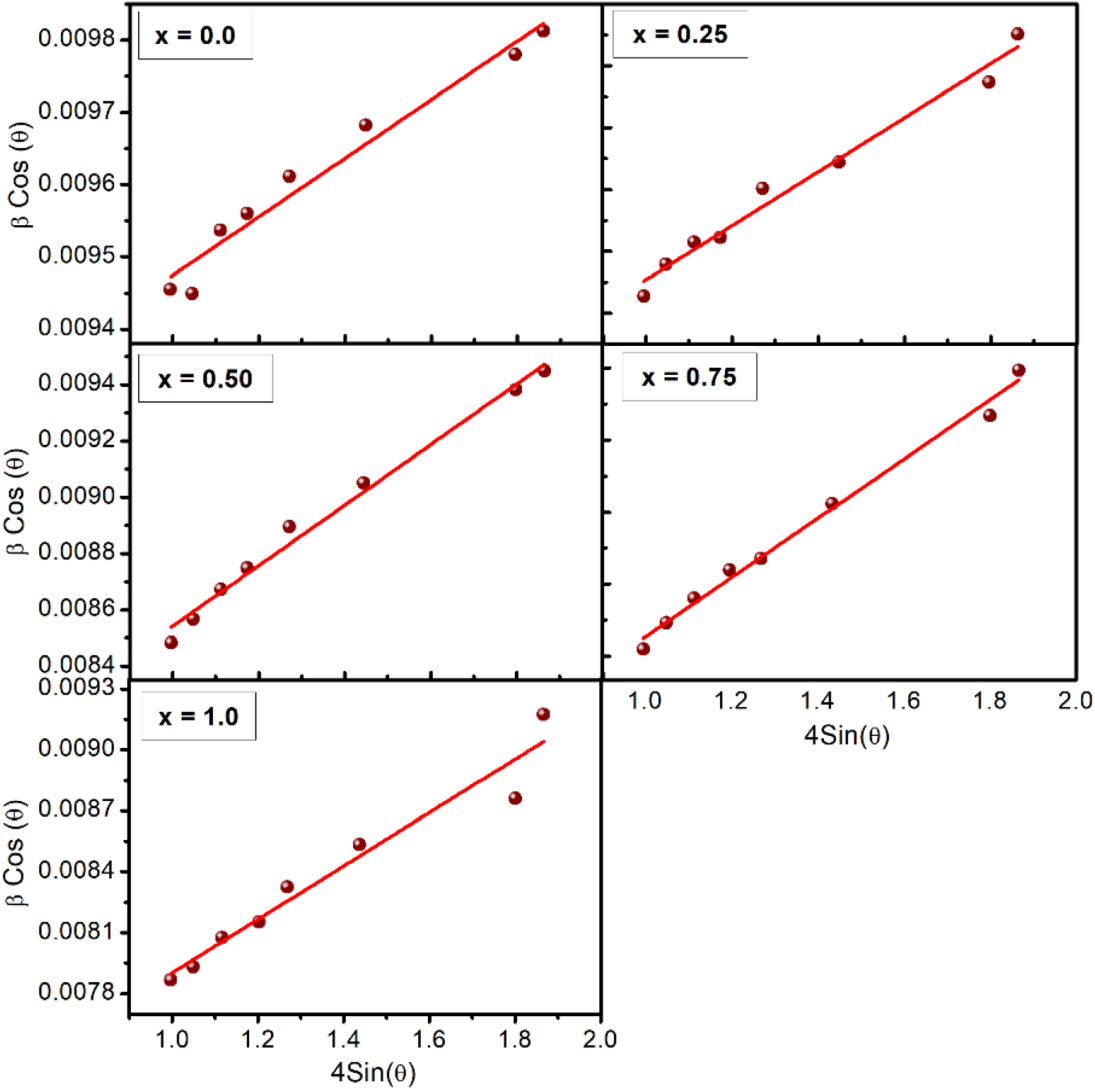
Williamson–Hall plots of Ba_0.5_Sr_0.5_Y_1.0_Fe_11−*x*_Co_*x*/2_Al_*x*/2_O_19_ samples.

To decouple the effects of crystallite size and lattice-induced microstrain on X-ray peak broadening, the Williamson–Hall (W–H) method was employed for all compositions of Ba_0.5_Sr_0.5_Y_1.0_Fe_11−*x*_(Co_*x*/2_Al_*x*/2_)O_19_ (*x* = 0.00–1.00). The corresponding W–H plots are presented in Fig. 2, wherein the modified full width at half maximum (FWHM) term *β* cos *θ* is plotted against 4 sin *θ*. The linear nature of these plots validates the underlying assumption that both crystallite size and strain contribute to the peak broadening, in accordance with the uniform deformation model (UDM). The W–H analysis is based on the following equation:^40^4
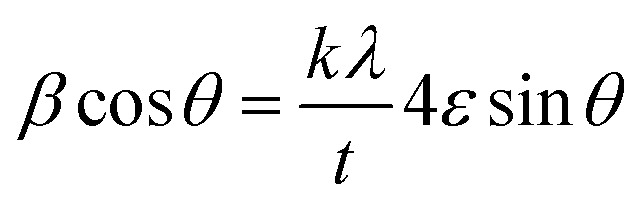
where *β* is the FWHM (in radians), *θ* is the Bragg angle, *k* is the shape factor (assumed as 0.9), *λ* is the wavelength of Cu Kα radiation (1.5406 Å), *t* is the effective crystallite size, and *ε* represents the lattice strain. From the linear fit of each W–H plot, the *y*-intercept yields the crystallite size, and the slope yields the strain component. The results, summarized in Table 3, reveal a consistent increase in both crystallite size and strain with increasing Co–Al substitution. The W–H-derived crystallite size increases from 17.75 nm for the undoped sample (*x* = 0.00) to 22.10 nm at *x* = 1.00. This trend is consistent with the Scherrer-based estimation, though the values are marginally higher due to the strain component being explicitly accounted for in W–H analysis. The increment in crystallite size suggests improved atomic diffusion and grain growth during high-temperature sintering, facilitated by the substituted elements, possibly due to their catalytic influence on grain boundary mobility.^41^

More significantly, the lattice strain (*ε*) shows a marked increase across the substitution series, ranging from 6.75 × 10^−4^ (*x* = 0.00) to 13.1 × 10^−4^ (*x* = 1.00). This nearly two-fold increase is attributed to the lattice distortion introduced by the ionic size mismatch between the host Fe^3+^ ions and the substituents Co^2+^ and Al^3+^. The random distribution of these substituent ions across the Fe sites likely introduces localized compressive and tensile stresses, leading to a net increase in microstrain throughout the crystal lattice.

The increase in lattice strain with substitution is an important structural response that may influence the magnetic anisotropy, domain wall pinning, and dielectric loss behavior of the material. It reflects a shift toward a more distorted lattice configuration, which can impact the Fe–O–Fe bond angles and lengths, thereby modifying the superexchange interactions fundamental to the magnetic properties of hexaferrites. Moreover, elevated lattice strain may contribute to enhanced dielectric relaxation and energy dissipation, crucial for microwave absorption applications.

**Fig. 3:**
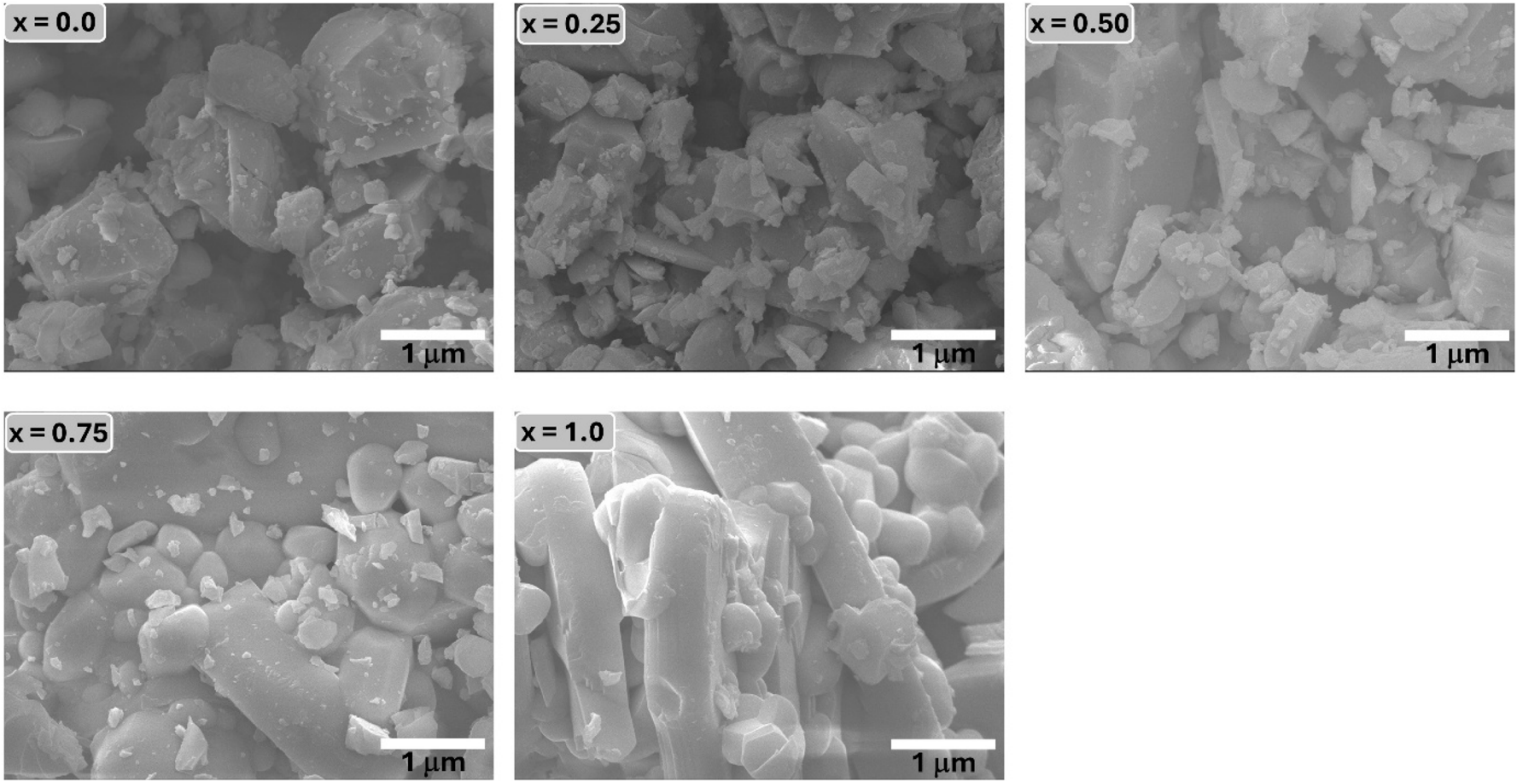
Field emission scanning electron microscopy (FESEM) images of all the samples of Ba_0.5_Sr_0.5_Y_1.0_Fe_11−*x*_Co_*x*/2_Al_*x*/2_O_19_.

The surface morphology and microstructural evolution of the Ba_0.5_Sr_0.5_Y_1.0_Fe_11−*x*_(Co_*x*/2_Al_*x*/2_)O_19_ were examined using FESEM, and the corresponding images are shown in Fig. 3. All samples exhibit tightly packed grains with noticeable agglomeration, confirming their microcrystalline nature. The particles display pseudo-spherical to platelet-like shapes, which are characteristic of M-type hexaferrites synthesized *via* sol–gel auto-combustion followed by high-temperature calcination.

As noted by the reviewer, the observed grain sizes lie in the micron range, which is expected for ferrites calcined at 1200 °C. Due to extensive agglomeration, overlapping grains, and irregular platelet morphologies, the SEM images do not permit reliable quantitative particle-size extraction; therefore, particle-size histograms are not presented, as they would not accurately represent the true size distribution. Instead, microstructural evolution is discussed qualitatively.

For the pristine sample (*x* = 0.00), the grains appear relatively small, porous, and loosely connected, indicating limited grain growth. With increasing Co–Al substitution, the grains become more consolidated and exhibit moderate coarsening, consistent with the crystallite-size trends obtained from XRD. The microstructures for *x* = 0.50 and 0.75 show improved uniformity, reduced porosity, and smoother particle surfaces, suggesting enhanced sintering behavior. At *x* = 1.00, elongated platelet-like grains and more pronounced agglomeration are observed, likely due to increased grain–grain interactions at higher substitution levels.

**Fig. 4:**
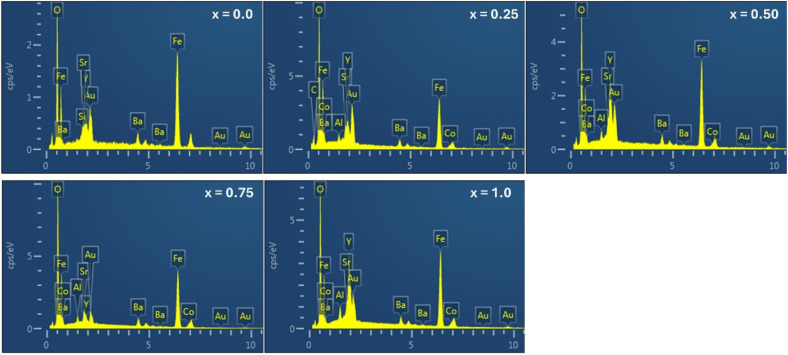
EDAX spectra of all the samples of Ba_0.5_Sr_0.5_Y_1.0_Fe_11−*x*_Co_*x*/2_Al_*x*/2_O_19_.

The elemental composition of Ba_0.5_Sr_0.5_Y_1.0_Fe_11−*x*_(Co_*x*/2_Al_*x*/2_)O_19_ (*x* = 0.00–1.00) was examined using SEM-EDX, and the corresponding spectra for all compositions are shown in Fig. 4. For the pristine sample (*x* = 0.00), the EDAX spectrum exhibits strong peaks corresponding to Fe, Ba, Sr, Y, and O, in excellent agreement with the nominal stoichiometry. Upon introducing Co and Al (*x* ≥ 0.25), additional peaks attributed to the substituted ions appear and increase progressively in intensity with increasing substitution content, confirming their successful incorporation into the hexaferrite lattice. As expected, Fe peaks remain dominant but gradually decrease in relative intensity with higher *x*, reflecting the systematic replacement of Fe^3+^ by Co^2+^ and Al^3+^ ions.

The persistent presence and nearly constant wt% values of Ba, Sr, and Y across all compositions indicate that the A-site framework remains chemically stable during Co–Al substitution. Oxygen peaks remain strong and consistent throughout, suggesting that no significant oxygen deficiency or change in oxidation state is induced during synthesis. The appearance of gold (Au) peaks in all spectra originates from sputter-coating applied prior to FESEM imaging and does not represent actual sample chemistry.

Quantitative EDX data, summarized in Table 4, compare the theoretical and experimental weight percentages of all constituent elements. The experimental wt% values follow the expected compositional trends: Fe decreases with *x*, Co and Al increase proportionally, and Ba/Sr/Y remain essentially unchanged. The small deviations between theoretical and measured values are within the typical accuracy limits of EDX analysis and arise from factors such as peak overlaps (Fe–Co, Ba–Sr), surface topography, and the lower detection efficiency for light elements like oxygen. Importantly, no extraneous peaks associated with impurity phases or secondary oxides were detected, verifying the high chemical purity of the synthesized samples.

**Table 4:** Comparison of the theoretical and experimental weight percentages (wt%) of elements in Ba_0.5_Sr_0.5_Y_1_Fe_11–*x*_(Co_*x*/2_Al_*x*/2_)O_19_ (*x* = 0.00–1.00) obtained from EDX analysis

Element	*x* = 0.00		*x* = 0.25		*x* = 0.50		*x* = 0.75		*x* = 1.00	
	Theor.	Exp.	Theor.	Exp.	Theor.	Exp.	Theor.	Exp.	Theor.	Exp.
Ba	6.13	6.10	6.15	6.14	6.17	6.16	6.19	6.18	6.20	6.20
Sr	3.91	3.88	3.93	3.90	3.94	3.92	3.95	3.93	3.96	3.95
Y	7.94	7.90	7.96	7.92	7.99	7.94	8.01	7.96	8.03	7.98
Fe	54.86	55.20	54.08	54.27	53.30	53.60	52.54	52.80	50.46	50.90
Co	0.00	0.00	0.62	0.61	1.23	1.19	1.84	1.83	2.66	2.58
Al	0.00	0.00	0.28	0.26	0.56	0.54	0.84	0.82	1.22	1.15
O	27.15	26.92	26.98	26.90	26.80	26.65	26.63	26.48	27.47	27.20

These observations confirm the chemical homogeneity and effective elemental incorporation achieved through the sol–gel auto-combustion method. The excellent agreement between theoretical and experimental wt% values provides strong compositional validation and directly supports the cation distribution model proposed in Table 4, reinforcing the structural inferences drawn from Rietveld refinement. Overall, the EDX analysis confirms that Co–Al substitution has been chemically realized without introducing secondary phases, thereby preserving the integrity of the M-type hexaferrite system for subsequent magnetic and dielectric investigations.

**Fig. 5:**
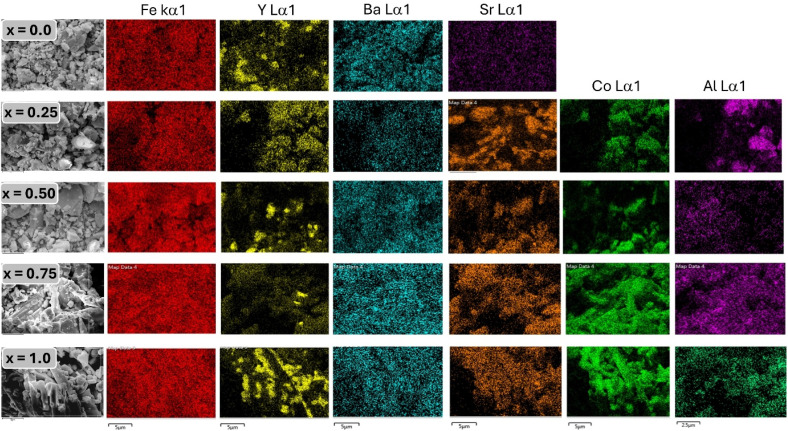
Color elemental mapping of Ba_0.5_Sr_0.5_Y_1.0_Fe_11−*x*_Co_*x*/2_Al_*x*/2_O_19_ samples.

To further confirm the spatial distribution and homogeneity of constituent elements in Ba_0.5_Sr_0.5_Y_1.0_Fe_11−*x*_(Co_*x*/2_Al_*x*/2_)O_19_, energy-dispersive X-ray spectroscopy (EDS) elemental mapping was performed, and the results are shown in Fig. 5 for all substitution levels (*x* = 0.00 to 1.00). Each row of images represents a sample with a specific substitution level, while the columns display elemental maps for Fe, Y, Ba, Sr, Co, and Al, superimposed on corresponding SEM micrographs.

In the unsubstituted sample (*x* = 0.00), strong and uniform elemental signals are observed for Fe, Ba, Sr, and Y, with no signals from Co or Al, confirming the stoichiometric formation of the base hexaferrite phase. The elemental distribution appears homogeneous across the scanned area, indicating efficient mixing and successful gel combustion during synthesis. With the introduction of Co and Al at *x* = 0.25, both elements begin to appear in the elemental maps. Notably, Co (orange) and Al (purple) distributions are visibly uniform, showing no evidence of elemental segregation, clustering, or localized enrichment. As the substitution level increases through *x* = 0.50 and 0.75, the Co and Al signals intensify proportionally, further validating their systematic incorporation into the hexaferrite matrix. Importantly, the elemental maps for Fe exhibit a complementary decrease in intensity, consistent with the gradual replacement of Fe^3+^ by Co^2+^ and Al^3+^ ions. At the highest substitution level (*x* = 1.00), all elements including Fe, Ba, Sr, Y, Co, and Al continue to display excellent spatial homogeneity. This uniform elemental dispersion reflects high phase purity and successful chemical incorporation of dopants without phase separation or formation of unwanted secondary phases. Such uniformity is critical for maintaining magnetic and dielectric uniformity across the material, as compositional inhomogeneity can lead to localized strain fields, magnetic anisotropy fluctuations, and dielectric scattering centers.

**Fig. 6:**
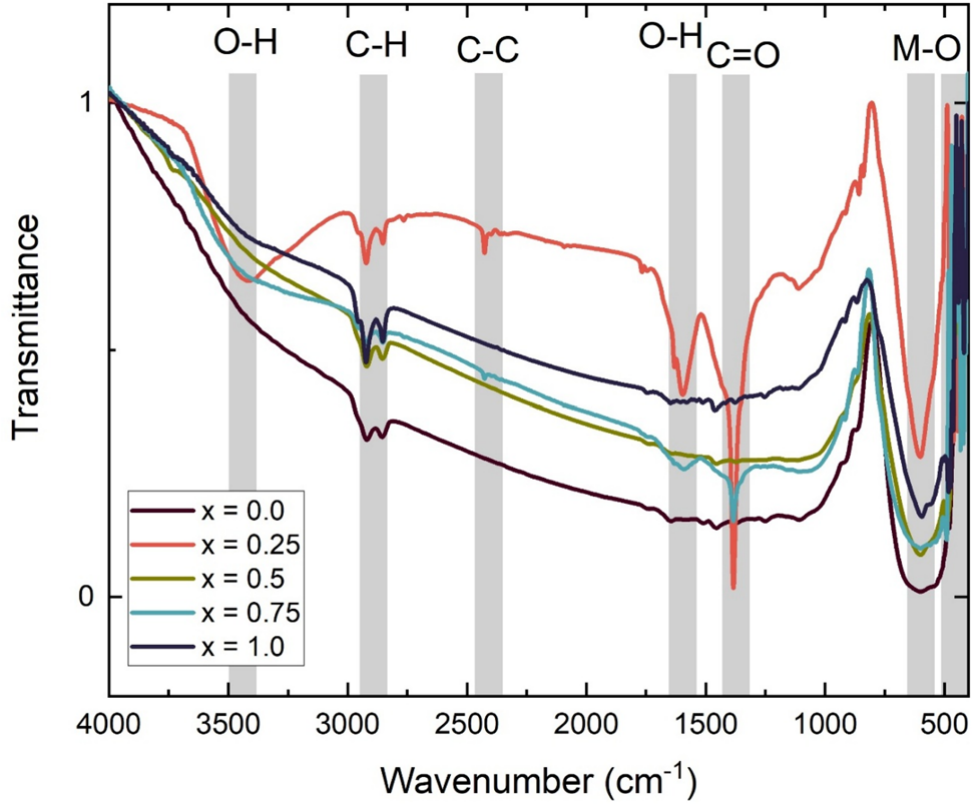
FTIR spectra of Ba_0.5_Sr_0.5_Y_1.0_Fe_11−*x*_Co_*x*/2_Al_*x*/2_O_19_.

The structural integrity and vibrational characteristics of the Ba_0.5_Sr_0.5_Y_1.0_Fe_11−*x*_(Co_*x*/2_Al_*x*/2_)O_19_ hexaferrite system were further explored using Fourier-transform infrared (FTIR) spectroscopy in the range of 4000–400 cm^−1^. The FTIR spectra, presented in Fig. 6, exhibit multiple absorption bands corresponding to molecular vibrations of functional groups and characteristic lattice vibrations of metal–oxygen (M–O) bonds. These bands provide crucial insight into the phase formation and local structural environment of the synthesized materials.^42^

In the high-wavenumber region, a broad band centered around 3400 cm^−1^ is consistently observed for all compositions. This band is attributed to O–H stretching vibrations, indicating the presence of adsorbed water molecules or surface hydroxyl groups. The accompanying bending vibration of H–O–H near 1620 cm^−1^ further confirms this assignment. Bands in the range 2800–3000 cm^−1^ are due to C–H and C–C stretching modes, while weak signals between 1400–1700 cm^−1^ may be associated with carboxylate (C=O) groups, likely residues from citric acid precursors used during the sol–gel process.^43,44^ The gradual attenuation of these organic-related bands with increasing *x* suggests more complete combustion of organics and improved crystallization in Co–Al substituted samples. The most significant features appear in the low-wavenumber region (400–700 cm^−1^), which correspond to M–O stretching vibrations in both tetrahedral and octahedral sites characteristic of the M-type hexaferrite structure. Two principal peaks in this region are denoted as *ν*_1_ and *ν*_2_, and their exact positions were extracted from the experimental spectra.

For the unsubstituted sample (*x* = 0.00), the *ν*_1_ and *ν*_2_ bands are observed at 599.90 cm^−1^ and 479.10 cm^−1^, respectively. These are attributed to Fe–O stretching in octahedral and tetrahedral coordination. With progressive substitution of Co^2+^ and Al^3+^ for Fe^3+^, subtle shifts in the band positions are observed. At *x* = 0.25 and *x* = 0.50, *ν*_1_ remains unchanged at 599.90 cm^−1^, but *ν*_2_ shifts to 509.81 cm^−1^ and 489.34 cm^−1^, respectively, suggesting that the local force constants in tetrahedral sites are altered due to lattice strain or ionic radius effects introduced by the dopants. Interestingly, at higher substitution levels (*x* = 0.75 and *x* = 1.00), both bands shift more noticeably. For *x* = 0.75, *ν*_2_ increases significantly to 567.14 cm^−1^, while *ν*_1_ remains at 599.90 cm^−1^. At *x* = 1.00, *ν*_1_ slightly shifts to 593.75 cm^−1^, while *ν*_2_ remains at 567.14 cm^−1^. These shifts reflect increasing distortion of the metal–oxygen framework, likely arising from ionic size mismatch (Fe^3+^*vs.* Co^2+^ and Al^3+^) and altered bond covalency. Co^2+^, having unfilled d-orbitals and larger polarizability, may enhance M–O bond strength, while Al^3+^, being smaller and more electronegative, introduces lattice stiffening effects.^45^

The systematic variation in *ν*_1_ and *ν*_2_ band positions with increasing substitution provides strong vibrational evidence of successful incorporation of dopant ions into the hexaferrite lattice. These changes are consistent with the observed lattice strain and contraction from XRD analysis and reflect the evolving local symmetry and bond environment critical to magnetic exchange interactions and dielectric polarization behavior.

### 3.2 Magnetic properties

**Fig. 7:**
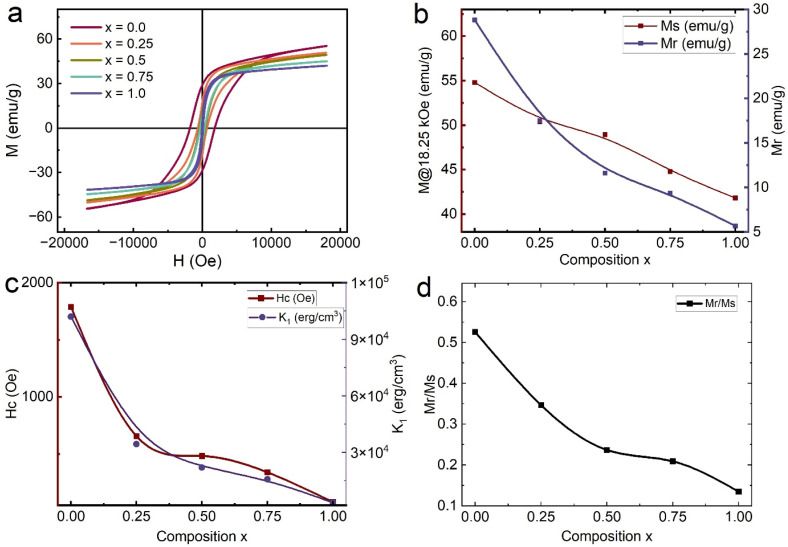
Magnetic characterization of Ba_0.5_Sr_0.5_Y_1.0_Fe_11−*x*_Co_*x*/2_Al_*x*/2_O_19_ (*x* = 0.0–1.0): (a) M–H hysteresis loops measured at room temperature; (b) variation of magnetization at 18.25 kOe; and variation of remanent magnetization; (c) coercivity (*H*_c_); and (d) remanent ratio (*M*_r_/*M*_s_) as functions of Co–Al substitution.

The magnetic behavior of Ba_0.5_Sr_0.5_Y_1.0_Fe_11−*x*_(Co_*x*/2_Al_*x*/2_)O_19_ (*x* = 0.00–1.00) hexaferrite particles was systematically studied using vibrating sample magnetometry at room temperature. The measured M–H hysteresis loops for all compositions are shown in Fig. 7a, while compositional variations in saturation magnetization (*M*_s_), remanent magnetization (*M*_r_), coercivity (*H*_c_), remanence ratio (*M*_r_/*M*_s_), and magnetocrystalline anisotropy constant (*K*_1_) are plotted in Fig. 7b–d.

The pristine Ba_0.5_Sr_0.5_Y_1.0_Fe_11_O_19_ sample exhibits a high saturation magnetization of 54.79 emu g^−1^, characteristic of M-type hexaferrites where Fe^3+^ ions (5*µ*^B^) occupy five distinct crystallographic sites (12k, 2a, 4f_2_, 4f_1_, and 2b), distributed between spin-up and spin-down sublattices. The net magnetic moment results from superexchange interactions between Fe^3+^ ions *via* O^2−^ bridges, primarily of the type: Fe^3+^(Oh)–O^2−^–Fe^3+^(Td).

Upon substitution, Co^2+^ (d^7^, 3*µ*^B^ in high spin) and Al^3+^ (d^0^, 0*µ*^B^) ions replace Fe^3+^, thereby disrupting these superexchange pathways. As *x* increases from 0.00 to 1.00, *M*_s_ declines to 41.80 emu g^−1^, indicating a dilution of the magnetic sublattice. This drop can be attributed to: (i) reduced number of Fe^3+^–Fe^3+^ coupling pairs, (ii) introduction of non-magnetic Al^3+^ into spin-down sites, and (iii) partial replacement by Co^2+^, which prefers different crystallographic environments and contributes lower magnetic moments than Fe^3+^.

The substitution likely favors occupancy of 4f_1_ and 4f_2_ spin-down sites by Al^3+^, reducing the net magnetic moment due to their antiferromagnetic coupling nature. Furthermore, local structural distortion due to ionic size mismatch (Fe^3+^: 0.645 Å; Al^3+^: 0.535 Å; Co^2+^: 0.72 Å) may induce canting of magnetic spins and increase spin disorder at grain boundaries.

The remanent magnetization (*M*_r_) follows a similar trend, decreasing from 28.81 emu g^−1^ (*x* = 0.0) to 5.66 emu g^−1^ (*x* = 1.0), reflecting diminished domain alignment stability after removal of the external magnetic field. This is further captured by the remanence ratio (*M*_r_/*M*_s_), a measure of magnetic squareness, which declines from 0.526 to 0.135 with increasing substitution (Fig. 7d). The low *M*_r_/*M*_s_ values at higher *x* indicate transition toward a multi-domain or superparamagnetic-like state, where thermal fluctuations and reduced anisotropy weaken remanent alignment.

Coercivity is strongly dependent on both magnetocrystalline anisotropy (*K*_1_) and microstructural factors such as grain size, porosity, and domain wall pinning. In uniaxial hexaferrites, the relationship between coercivity and anisotropy is approximately given by:5
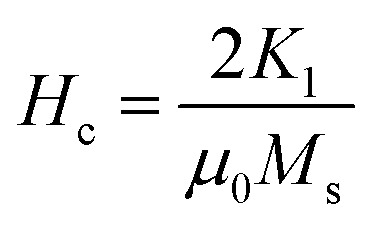
*H*_c_ is coercivity, *K*_1_ is the first-order magnetocrystalline anisotropy constant, *µ*_0_ is the permeability of free space, *M*_s_ is saturation magnetization.

For the unsubstituted sample (*x* = 0.0), *H*_c_ = 1788.8 Oe, consistent with strong uniaxial anisotropy (*K*_1_ = 1.02 × 10^5^ erg cm^−3^), which originates from the spin–orbit coupling of Fe^3+^ ions occupying high-anisotropy sites like 2b. As substitution increases, *H*_c_ decreases sharply to 77.7 Oe for *x* = 1.0, and *K*_1_ concurrently drops to 3.38 × 10^3^ erg cm^−3^ (Fig. 7c). This dramatic softening of magnetic behavior results from the dilution of anisotropic Fe^3+^ centers by Co^2+^ and Al^3+^. Al^3+^, in particular, being non-magnetic and spherically symmetric (d^0^), significantly reduces local anisotropy and introduces spin disorder.

Moreover, Co^2+^ substitution is known to prefer low-anisotropy sites in the spin-down sublattice (*e.g.*, 4f_1_), which contributes less to magnetic hardness. The combined ionic substitutions thus reduce effective *K*_1_ and eliminate domain wall pinning centers, enhancing domain wall mobility and resulting in low coercivity.

The magnetocrystalline anisotropy constant *K*_1_ was estimated using the law:6
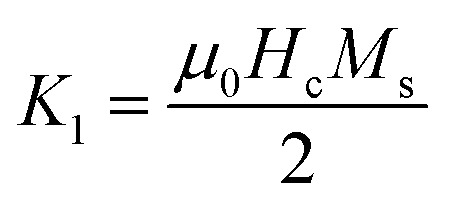
Values extracted from this relation show a monotonic decline with substitution, confirming the loss of intrinsic magnetic anisotropy. This has profound implications on the magnetic performance at high frequency, where high *K*_1_ is essential to suppress eddy currents and maintain permeability stability.

The progressive magnetic softening observed across the Ba_0.5_Sr_0.5_Y_1.0_Fe_11−*x*_(Co_*x*/2_Al_*x*/2_)O_19_ series can be attributed to several synergistic mechanisms. Primarily, the substitution of Fe^3+^ ions with non-magnetic Al^3+^ and weakly magnetic Co^2+^ leads to a net reduction in the number of magnetically active ions, thereby weakening the superexchange interactions responsible for long-range ferrimagnetic order. This reduction in Fe^3+^ content not only decreases the saturation magnetization but also disrupts the delicate balance between spin-up and spin-down sublattices, leading to spin dilution and symmetry breaking. Furthermore, the introduction of Al^3+^ which possesses a d^0^ electronic configuration,^46^ reduces anisotropy primarily through its occupation of the 4f_1_ and 4f_2_ sites, weakening the spin-down sublattice contributions. Co^2+^, in accordance with site energetics, predominantly occupies the 12k octahedral sites and contributes to localized anisotropy modification. Simultaneously, structural analyses reveal an increase in microstrain (*ε*) with substitution, as confirmed by Williamson–Hall plots, which introduces additional local lattice distortions that further destabilize spin alignment. The combined effects of these factors compositional dilution, anisotropy suppression, site-specific substitution, and strain-induced magnetic disorder drive the transition of the material from a magnetically hard phase (*x* = 0.0) suitable for permanent magnet applications to a magnetically soft phase (*x* = 1.0), potentially more favorable for applications requiring low coercivity, such as electromagnetic interference (EMI) shielding, transformer cores, and broadband microwave absorbers.

### 3.3 Microwave absorption properties

**Fig. 8:**
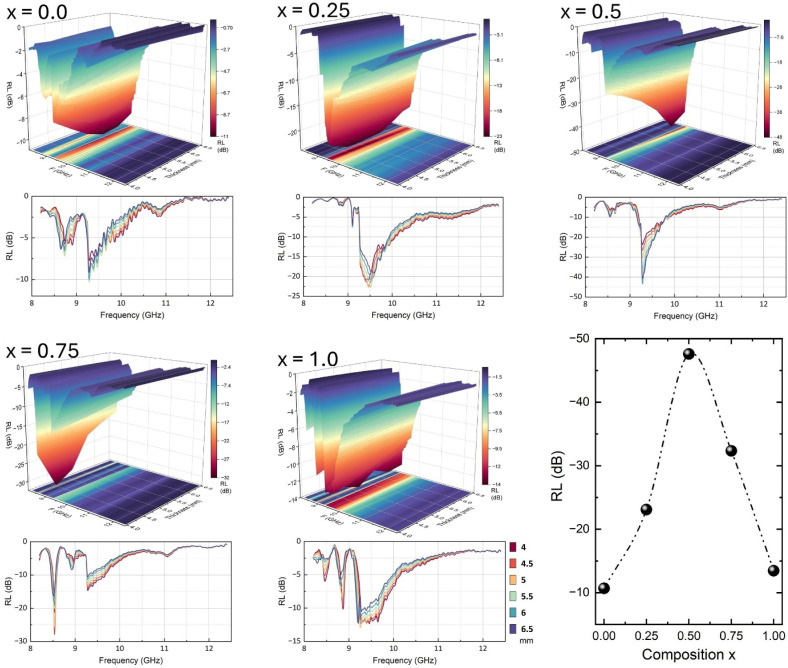
Microwave absorption properties of Ba_0.5_Sr_0.5_Y_1.0_Fe_11−*x*_Co_*x*/2_Al_*x*/2_O_19_ (*x* = 0.0–1.0): reflection loss (RL) as a function of frequency in the X-band (8–12 GHz). The bottom right shows the variation of minimum RL values with substitution level *x*.

The microwave absorption characteristics of Ba_0.5_Sr_0.5_Y_1.0_Fe_11−*x*_(Co_*x*/2_Al_*x*/2_)O_19_ (*x* = 0.00–1.00) hexaferrite composites were evaluated in the X-band region (8–12 GHz), with the reflection loss (RL) behavior plotted in Fig. 8. The performance metrics minimum reflection loss (RL_min_), matching frequency, optimal thickness, and effective absorption bandwidth (EAB) are detailed using both experimental plots and quantitatively extracted values from the microwave absorption dataset, are summarized in Table 5.

**Table 5:** Key microwave absorption performance metrics Ba_0.5_Sr_0.5_Y_1.0_Fe_11−*x*_Co_*x*/2_Al_*x*/2_O_19_ in the X-band (8–12 GHz), detailing minimum reflection loss (RL_min_), frequency, thickness and maximum effective absorption bandwidth (EAB, defined by RL ≤−10 dB)

Sample	*f* (GHz)	Thickness (mm)	RL (dB)	EAB (GHz)
*x* = 0.0	9.271	5.5	−10.68	0.021
*x* = 0.25	9.46	5	−23.08	0.756
*x* = 0.5	9.271	6	−47.58	0.609
*x* = 0.75	8.536	4.5	−32.33	0.378
*x* = 1.0	9.25	5	−13.47	0.441

The unsubstituted sample (*x* = 0.0) exhibits an RL_min_ of −10.68 dB at 9.27 GHz for a matching thickness of 5.5 mm, corresponding to about 90% power attenuation. However, the effective absorption bandwidth is narrow, spanning only 0.021 GHz in the 9.271–9.292 GHz range. With the introduction of Co–Al substitution, significant improvements are observed in both RL depth and bandwidth. At *x* = 0.25, RL_min_ drops to −23.08 dB at 9.46 GHz (5 mm thickness), and the EAB dramatically increases to 0.756 GHz, covering the range 9.271–10.027 GHz, making it suitable for broader X-band applications.

The optimal absorption performance is recorded at *x* = 0.50, where the RL_min_ reaches −47.58 dB at 9.27 GHz with a thickness of 6 mm. This reflects nearly 100% microwave attenuation, and the extracted EAB spans 0.609 GHz, distributed across two segments: 9.166–9.775 GHz and a narrow point at 9.838 GHz. This multi-peak profile suggests the simultaneous presence of multiple loss mechanisms, possibly arising from hybrid dielectric–magnetic loss resonance and improved impedance matching. At higher substitution (*x* = 0.75), the RL_min_ slightly decreases to −32.33 dB, but the EAB is fragmented into three distinct bands 8.515–8.578 GHz, 9.271–9.586 GHz, and a single-point absorption at 9.649 GHz totaling 0.378 GHz. While absorption remains strong, the fragmentation indicates complex wave–material interactions potentially arising from localized inhomogeneities or over-substitution effects. At *x* = 1.0, a further reduction in RL_min_ to −13.47 dB is observed at 9.25 GHz (5 mm thickness), with an EAB of 0.441 GHz, spanning the range 9.229–9.670 GHz. Although this still qualifies as moderate absorber performance, the decline compared to *x* = 0.50 is attributed to excessive substitution leading to magnetic dilution and a breakdown in the impedance-matching condition. The reflection loss behavior follows the fundamental transmission line model:7
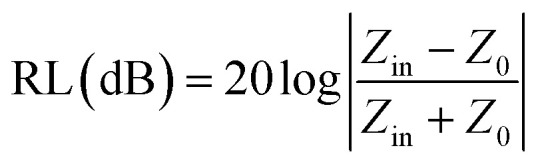
where the input impedance *Z*_in_ of the absorber is governed by:8
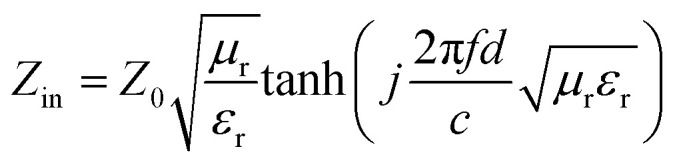
In this framework, efficient microwave absorption depends on achieving impedance matching (*Z*_in_ ≈ *Z*_0_) along with substantial intrinsic losses (from *µ*″ and *ε*″). In the present system, substitution with Co^2+^ enhances magnetic losses through mechanisms such as spin relaxation and ferromagnetic resonance, while Al^3+^, being non-magnetic, modulates the dielectric constant and introduces interfacial polarization at grain boundaries. Furthermore, the combined effect of substitution and microstructural refinement (reduced porosity, improved connectivity, optimized particle size) enhances both dielectric and magnetic loss tangents, contributing to broader and deeper RL profiles.

Thus, Co–Al dual substitution proves to be a powerful tuning parameter for optimizing microwave absorption in hexaferrite-based composites. The composition *x* = 0.50 emerges as the best-performing sample, offering near-complete absorption at relatively moderate thickness with a wide bandwidth suitable for radar stealth and EMI suppression technologies. The ability to tailor EAB and RL characteristics by compositional design opens avenues for developing high-efficiency, frequency-selective absorbers in GHz regimes.

A comparative analysis of our results with previously reported doped M-type hexaferrites is presented in Table 6. The comparison reveals that while many substituted hexaferrite systems exhibit either strong microwave absorption or enhanced magnetic performance, it is uncommon for a single material system to simultaneously achieve both attributes to a significant extent.

**Table 6:** Comparison of magnetic and microwave absorption performance of hexaferrite materials

Sample	*M*_s_ (emu g^−1^)	*M*_r_/*M*_s_	*H*_c_ (Oe)	*t* (mm)	*R*_L_ (dB)	Absorption frequency (GHz)	Ref.
Ba_0.5_Ca_0.5_Dy_0.10_Fe_11.90_O_19_	96.5	0.4	1146	3	−26.96	10.06	47
BaCe_0.75_Dy_0.75_Fe_10.5_O_19_	26.3	0.59	5721	3	−16.3	19.72	18
Sr(Zr–Mn)_2*x*_Fe_12−2*x*_O_19_	59	0.52	2384	2	−27.68	10.14	48
Ba(Mn_0.5_Co_0.5_Ti)_0.5_Fe_11_O_19_	67.05	0.22	3107	2.3	−27.4	12.34	49
BaCu_*x*_Mg_*x*_Zr_2*x*_Fe_12−4*x*_O_19_	50	05	4420	3.3	−14.4	9	50
BaFe_12−2*x*_Co_*x*_Zn_*x*_O_19_	23	0.65	2890	1	−29.98	10.8	51
Ba_0.5_Sr_0.5_Co_*x*_Ga_*x*_Fe_12−2*x*_O_19_	59.6	0.31	800	2.0	−29.74	8.28	52
Ba_(1−2*x*)_La_*x*_Na_*x*_Fe_10_CoTiO_19_	77.4	0.28	361	1.4	−29.4	24.6	53
BaFe_12−*x*_Al_*x*_O_19_	66.95	0.51	5589	3	−34.74	14.3	54 and 55
BaFe_12−*x*_Cr_*x*_O_19_	67.08	0.52	3821	3	−27	9.7	
Ba_0.5_Sr_0.5_Y_1.0_Fe_11−*x*_Co_*x*/2_Al_*x*/2_O_19_	54.79	0.526	1788	6	−47.58	9.27	This work

### 3.4 Dielectric properties

**Fig. 9:**
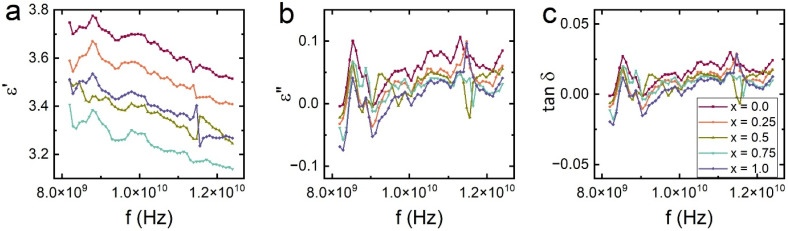
Frequency-dependent dielectric properties of Ba_0.5_Sr_0.5_Y_1.0_Fe_11−*x*_Co_*x*/2_Al_*x*/2_O_19_ (*x* = 0.0–1.0) in the range of 8–12 GHz: (a) real part of dielectric constant (*ε*′), (b) dielectric loss (*ε*″) and (c) dielectric loss tangent (tan *δ*).

The dielectric response of Ba_0.5_Sr_0.5_Y_1.0_Fe_11−*x*_(Co_*x*/2_Al_*x*/2_)O_19_ composites over the X-band frequency range (8–12 GHz) is depicted in Fig. 9 in terms of real permittivity (*ε*′), imaginary permittivity (*ε*″), and dielectric loss tangent (tan *δ*). These properties are vital for understanding energy storage, dissipation, and the underlying polarization mechanisms governing microwave interactions in substituted hexaferrite systems.

The real part of permittivity (*ε*′) decreases with increasing frequency for all substitution levels, consistent with the general dielectric relaxation behavior where dipoles fail to follow the rapidly alternating external field. At low frequencies (8–9 GHz), *ε*′ values are relatively high, particularly for the pristine (*x* = 0.0) and lightly substituted (*x* = 0.25) samples. This elevated permittivity in the low-frequency region arises predominantly from Maxwell–Wagner interfacial polarization,^56,57^ as explained by Koops' two-layer model,^58^ which envisions the ceramic material as consisting of well-conducting grains separated by poorly conducting grain boundaries.

According to Koops' theory, the total dielectric constant is dominated by the grain boundaries at low frequencies and by grains at higher frequencies. In the present system, grain boundaries act as charge traps, accumulating space charge at interfaces. This interfacial polarization leads to high *ε*′ at low frequencies, which diminishes as frequency increases and the interfacial dipoles can no longer realign with the oscillating field. The microstructural observations from FESEM corroborate this behavior: the pristine and lightly doped samples exhibit larger grain boundaries and more heterogeneous microstructures, thus enhancing interfacial polarization.

As the Co–Al substitution increases, a progressive decrease in *ε*′ is observed across all frequencies, reaching minimum values for *x* = 1.0. This is due to the dual effects of:

(i) Reduced space charge density, as Al^3+^ (d^0^, non-polarizable) and Co^2+^ (low-field spin stabilizer) substitute Fe^3+^ and limit polaronic hopping (Fe^2+^/Fe^3+^), and

(ii) Densification of the microstructure, which narrows grain boundaries and thus diminishes interfacial polarization contributions.

The imaginary part of permittivity (*ε*″), which reflects dielectric losses arising from both conduction and polarization relaxation, also follows a decreasing trend with frequency. At lower substitution (*x* = 0.0 and *x* = 0.25), *ε*″ is relatively high due to strong conduction loss originating from hopping of localized electrons between Fe^2+^ ↔ Fe^3+^ sites. However, with increasing *x*, this hopping pathway is disrupted due to the replacement of Fe by non-conducting Al^3+^, leading to reduced loss and enhanced insulation. Additionally, substitution lowers the density of oxygen vacancies, another key contributor to conduction-related losses in ferrites.

These dielectric loss trends are consistent with the Debye-type relaxation model,^59,60^ expressed as:9
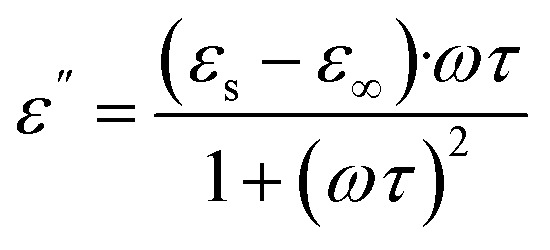
where *ε*_s_ is static permittivity, *ε*_∞_ the high-frequency limit, *ω* angular frequency, and *τ* the relaxation time. The flattening of *ε*″ spectra at higher substitution levels suggests broader and slower dielectric relaxation, possibly due to fewer active dipoles and increased structural ordering.

The dielectric loss tangent (tan *δ* = *ε*″/*ε*′) exhibits maximum values at lower substitution (*x* = 0.0 and 0.25), indicating dominant polarization and conduction losses. Interestingly, tan *δ* reaches a minimum at *x* = 0.50, coinciding with the optimal microwave absorption performance. This supports the idea that maximized dielectric loss is not always favorable for high absorption; instead, effective impedance matching and balance between dielectric and magnetic losses are critical. The lowered tan *δ* in the *x* = 0.50 composition also reflects improved crystallinity, minimized internal defects, and better grain connectivity—structural attributes that suppress excessive charge accumulation and energy dissipation *via* leakage currents.

The dielectric behavior of the Ba–Sr–Y–Fe hexaferrite system thus is governed by a frequency- and composition-dependent interplay of intrinsic dipolar relaxation, Maxwell–Wagner interfacial polarization, and Koops-type microstructural heterogeneity. The substitution of Co^2+^ and Al^3+^ systematically modulates the grain and boundary conductivities, dielectric loss pathways, and polarization stability. These changes lead to improved impedance matching, which when coupled with optimized magnetic losses, enhances microwave absorption across the X-band spectrum. Notably, the composition *x* = 0.50 presents an ideal compromise, exhibiting stable dielectric properties, reduced loss tangent, and favorable polarization characteristics, all conducive to high-efficiency electromagnetic attenuation.

**Fig. 10:**
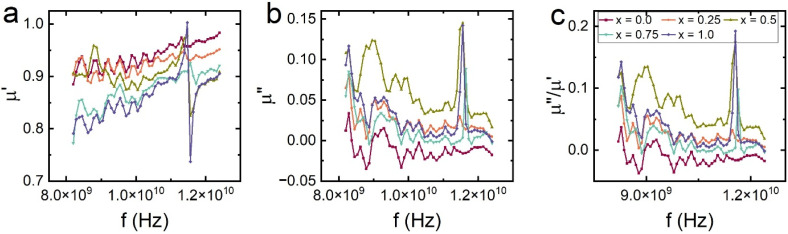
Frequency-dependent (a) real (*µ*′), (b) imaginary (*µ*″) and (c) *µ*″/*µ*′ part of permeability of Ba_0.5_Sr_0.5_Y_1.0_Fe_11−*x*_Co_*x*/2_Al_*x*/2_O_19_ (*x* = 0.0–1.0).

The frequency-dependent complex permeability of the Ba_0.5_Sr_0.5_Y_1.0_Fe_11−*x*_(Co_*x*/2_Al_*x*/2_)O_19_ system was examined in the 8–12 GHz X-band range and is presented in Fig. 10, comprising the real part of permeability (*µ*′) and the imaginary part (*µ*″). These components reflect the material's capacity to store and dissipate magnetic energy, respectively, and are critical for optimizing magnetic loss in microwave absorbing applications.

The real permeability (*µ*′) represents the reversible component of magnetization under an alternating magnetic field. For all compositions, *µ*′ exhibits a mild frequency dispersion, decreasing slowly with increasing frequency, a typical feature of ferrimagnetic materials governed by domain wall resonance and spin rotation. The unsubstituted sample (*x* = 0.00) displays the highest *µ*′ values, indicating strong magnetization response due to the high content of magnetically active Fe^3+^ ions occupying spin-aligned sublattices. As substitution with Co^2+^ and Al^3+^ progresses, *µ*′ gradually decreases. This decline is attributed to the dilution of Fe^3+^–O^2−^–Fe^3+^ superexchange interactions and reduced magnetic ordering caused by the introduction of non-magnetic Al^3+^ and weakly anisotropic Co^2+^ ions.

Importantly, Co^2+^ ions predominantly occupy the 12k sites, altering local anisotropy and magnetic damping without entering 2b or 4f_2_ positions. Meanwhile, Al^3+^ substitution contributes to magnetic dilution and suppresses *µ*′ by decreasing magnetic moment density and introducing non-magnetic centers that impede collective spin response.^61,62^ These modifications reduce the real permeability and shift the material toward magnetically softer behavior, consistent with the M–H and *K*_1_ trends discussed earlier.

The imaginary permeability (*µ*″), which represents magnetic energy loss due to lagging magnetization (hysteresis, spin relaxation, domain wall resonance), shows significant variation with frequency and composition. At *x* = 0.00, *µ*″ is relatively high at low frequencies, suggesting dominant domain wall contributions. However, as the frequency increases, *µ*″ drops, indicating limited resonance behavior in the GHz range. Upon Co–Al substitution, especially at *x* = 0.25 and *x* = 0.50, *µ*″ increases in the mid-X-band region, reflecting enhanced magnetic loss from resonance effects such as natural ferromagnetic resonance and eddy current dissipation.

The variation of the imaginary component of permeability (*µ*″) across the X-band frequency range highlights the presence of multiple magnetic loss mechanisms contributing to energy dissipation in the Ba_0.5_Sr_0.5_Y_1.0_Fe_11−*x*_(Co_*x*/2_Al_*x*/2_)O_19_ system. At lower substitution levels (*x* = 0.0 and 0.25), *µ*″ is elevated at lower frequencies, suggesting predominant contributions from domain wall motion. As frequency increases, however, the dominant loss mechanisms transition to high-frequency processes such as natural ferromagnetic resonance (FMR) and spin relaxation. Natural FMR arises from the precessional motion of magnetic moments in the presence of an internal anisotropy field,^63,64^*H*_k_, with the resonance frequency described by the equation:10
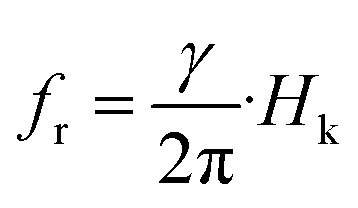
where *γ* is the gyromagnetic ratio. The substitution of Fe^3+^ by Co^2+^ and Al^3+^ alters the anisotropy field Hk, thereby shifting the FMR frequency and modulating the resonance behavior. Compositions with intermediate substitution, particularly *x* = 0.50, exhibit resonance-enhanced *µ*″ due to a favorable combination of anisotropy energy and magnetic damping, which promotes efficient magnetic energy absorption in the GHz regime.

In addition to FMR, spin relaxation becomes increasingly significant as Co^2+^ is incorporated into the lattice. Owing to its 3d^7^ electronic configuration, Co^2+^ can introduce strong spin–orbit coupling at specific lattice sites,^65,66^ resulting in localized magnetic anisotropy fluctuations. These fluctuations destabilize uniform spin precession and facilitate spin-lattice relaxation, thereby increasing magnetic loss at microwave frequencies. This effect is particularly pronounced in substituted samples with optimized Co content.

Another important contributor to magnetic loss is eddy current loss, which originates from the induction of circular currents within the magnetic grains by the time-varying magnetic field. This type of loss is typically frequency-dependent and scales with the square of the frequency and the electrical conductivity of the material. While eddy currents are suppressed in highly resistive or porous systems, they may remain appreciable in moderately dense ferrite composites where grain connectivity permits limited conduction. To assess the predominance of eddy current loss, the frequency dependence of the product *µ*″·*f*^−1^ (known as the *C* parameter) can be evaluated.^67^ A constant *C* value over the frequency range would suggest that eddy currents dominate the magnetic loss; however, deviations indicate a stronger contribution from resonance and relaxation mechanisms.

At higher substitution (*x* = 0.75 and *x* = 1.00), both *µ*′ and *µ*″ decrease, indicating suppression of both magnetic energy storage and loss. This is primarily due to excessive magnetic dilution and weakened anisotropy fields, which disrupt spin coherence and reduce magnetic damping capacity. The *µ*″ spectra become flatter, signifying diminished resonance activity and confirming the transition toward magnetically softer and lossy-diminished materials.

In totality, the complex permeability spectra reinforce the conclusion that *x* = 0.50 exhibits the most favorable magnetic response in the GHz range, offering a synergistic balance between *µ*′ and *µ*″. This contributes to enhanced microwave attenuation *via* magnetic loss mechanisms, particularly when coupled with dielectric matching. The tunable permeability *via* Co–Al substitution allows precise engineering of frequency-selective absorption behavior, making these materials suitable for targeted stealth and EMI applications.

**Fig. 11:**
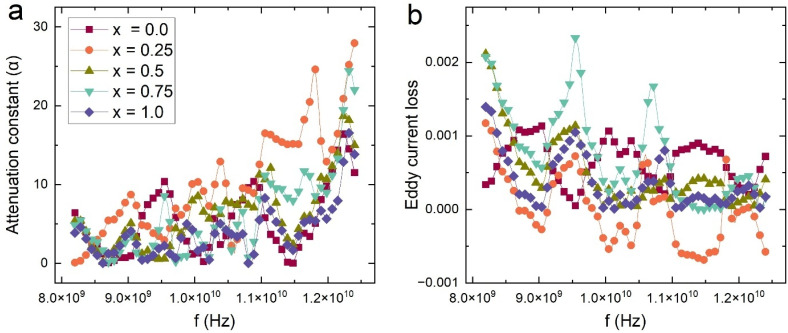
(a) Frequency-dependent attenuation constant (*α*) and (b) variation of eddy current with applied frequency of Ba_0.5_Sr_0.5_Y_1.0_Fe_11−*x*_(Co_*x*/2_Al_*x*/2_)O_19_ (*x* = 0.0–1.0) in the X-band region.

The electromagnetic wave attenuation characteristics of the Ba_0.5_Sr_0.5_Y_1.0_Fe_11−*x*_(Co_*x*/2_Al_*x*/2_)O_19_ system were further quantified through the calculation of the attenuation constant (*α*) and eddy current loss, both plotted in Fig. 11. These parameters provide crucial insights into the material's ability to absorb and dissipate incident electromagnetic energy *via* dielectric and magnetic losses. The attenuation constant (*α*), shown in Fig. 11a, reflects the degree to which the intensity of electromagnetic waves decays as they propagate through the material.^68^ It is derived using the complex permittivity and permeability values from the relation:11
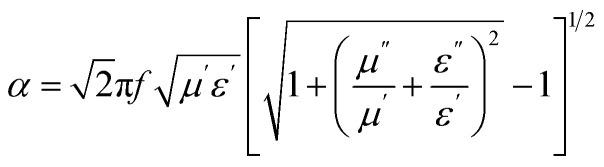


This expression accounts for both dielectric and magnetic contributions to the total loss. Across all compositions, *α* increases with frequency, particularly in the 10–12.5 GHz region, reflecting enhanced absorption capability at higher GHz frequencies due to stronger dipolar relaxation and magnetic resonance. Among all samples, the composition with *x* = 0.50 exhibits the highest *α* values over a broad frequency range, indicating its superior absorption efficiency. This trend aligns well with earlier findings from reflection loss (RL) and EAB analysis, where *x* = 0.50 also showed the widest bandwidth and deepest RL minimum.

The enhancement in *α* at *x* = 0.50 can be attributed to a combination of optimized magnetic loss (*µ*″), moderate dielectric loss (*ε*″), and effective impedance matching. As Co^2+^ and Al^3+^ substitute Fe^3+^, the reduction in magnetic anisotropy energy and increased spin relaxation at intermediate substitution create favorable conditions for electromagnetic wave attenuation. In contrast, the samples at *x* = 0.0 and *x* = 1.0 exhibit lower *α* values due to either excessive magnetic stiffness (*x* = 0.0) or over-dilution of magnetic centers and loss suppression (*x* = 1.0).

The eddy current loss, depicted in Fig. 11b, was estimated by plotting the frequency-dependent parameter *C*_0_ = *µ*″/*f*, which isolates the eddy current contribution from total magnetic loss. According to classical theory, if eddy current loss dominates, the *C*_0_ parameter should remain constant over the frequency range. However, in this case, the *C*_0_ curves vary significantly with frequency and composition, indicating that eddy current loss is not the dominant mechanism, especially in samples with lower conductivity and increased substitution.

At *x* = 0.0, a relatively flat *C*_0_ profile suggests minor eddy current activity due to the metallic nature of interconnected grains and residual Fe^3+^/Fe^2+^ redox pairs. However, at *x* = 0.50 and beyond, the *C*_0_ curves show non-uniform variation, suggesting that magnetic resonance and spin damping dominate the absorption behavior rather than eddy currents. The decline in *C*_0_ at high frequencies also implies that enhanced dielectric resistivity from Al^3+^ substitution and densified microstructure suppresses loop current formation.

Taken together, the attenuation constant and *C*_0_ analyses underscore the interplay of magnetic and dielectric losses in determining the microwave absorption performance of substituted hexaferrites. The data reveal that optimal substitution (*x* = 0.50) promotes multiple synergistic loss mechanisms including natural ferromagnetic resonance, domain wall damping, and dipolar relaxation while suppressing non-selective eddy losses, leading to efficient electromagnetic attenuation in the GHz regime.

### 3.5 Shielding effectiveness properties

**Fig. 12:**
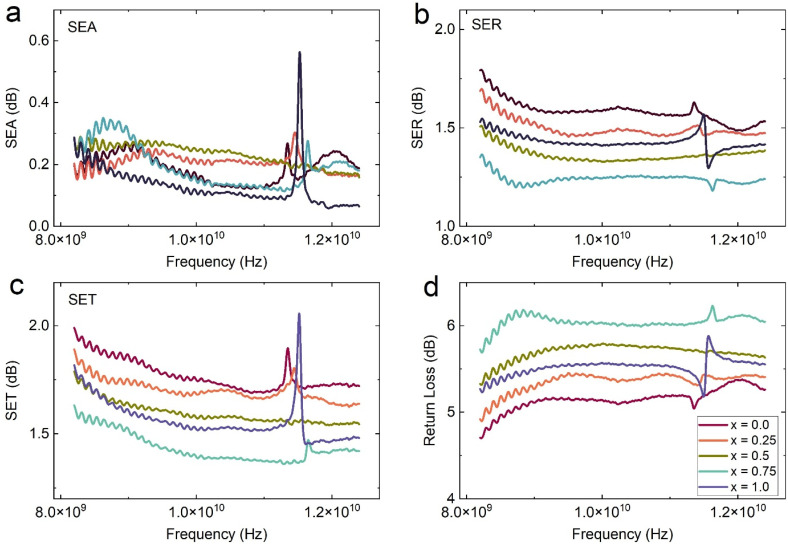
Variation of (a) shielding by absorption (SEA), (b) shielding by reflection (SER), (c) total shielding effectiveness (SET), and (d) return loss (RL) of all samples of Ba_0.5_Sr_0.5_Y_1.0_Fe_11−*x*_Co_*x*/2_Al_*x*/2_O_19_ (*x* = 0.0–1.0).

Fig. 12 presents the frequency-dependent electromagnetic shielding response of Ba_0.5_Sr_0.5_Y_1.0_Fe_11−*x*_(Co_*x*/2_Al_*x*/2_)O_19_ ferrite composites across the 8–12.5 GHz range. The shielding effectiveness (SE) has been resolved into three key components: shielding by absorption (SEA), shielding by reflection (SER), and total shielding effectiveness (SET).^69–71^ Additionally, return loss (RL) is plotted to evaluate the impedance matching and signal reflection behavior, which are critical for stealth and microwave absorbing materials.

In Fig. 12a, the SEA values for all compositions range between 0.15 and 0.60 dB, and notably increase with frequency due to stronger interaction between the material's dipoles and the oscillating electromagnetic field at higher GHz values. The *x* = 0.25 and *x* = 0.50 compositions exhibit higher SEA across the X-band, which aligns with their enhanced attenuation constant (*α*) and optimized dielectric–magnetic synergy. The elevated SEA in these compositions reflects greater microwave energy dissipation within the bulk of the material, primarily *via* dielectric relaxation, magnetic resonance, and interfacial polarization mechanisms.

Fig. 12b shows the SER component, which dominates the overall SE in this system, with values ranging from 1.2 to 1.9 dB. SER stems from the impedance mismatch at the material–air interface, leading to partial reflection of incident waves. The pristine sample (*x* = 0.0) exhibits the highest SER due to its elevated dielectric and magnetic contrast relative to free space. However, with Co–Al substitution, the SER decreases modestly, especially for *x* = 0.75, indicating improved impedance matching and reduced reflection. This transition enhances energy penetration into the material and complements the higher absorption noted in SEA for intermediate compositions.

The SET, depicted in Fig. 12c, is the cumulative effect of both SEA and SER, with values ranging from approximately 1.5 to 2.2 dB. The composition *x* = 0.0 records the highest SET, largely influenced by its high SER. However, from an absorber application standpoint, a high SEA-to-SET ratio is more desirable than a high total SET alone. In this context, *x* = 0.50 again emerges as optimal, offering a favorable balance between reflection suppression and absorption enhancement, which is more critical for non-reflective stealth technologies.

Complementing the shielding analysis, Fig. 12d presents the return loss (RL), a measure of reflected signal strength due to impedance mismatch. A higher RL corresponds to better absorption and lower reflection, signifying improved impedance matching with free space. The RL values increase progressively with substitution and reach a maximum for *x* = 0.75, followed closely by *x* = 0.50. This trend is coherent with earlier observations of the tan *δ* and *µ*″ spectra, where magnetic and dielectric losses were optimized for these substitution levels. Importantly, RL values exceeding 6 dB, as seen in *x* = 0.50 and *x* = 0.75, indicate that more than 75% of the incident microwave energy is attenuated, confirming their effectiveness as microwave absorbers.

Shielding performance is governed by a complex interplay between dielectric loss (interfacial polarization, Maxwell–Wagner relaxation), magnetic loss (domain wall resonance, natural ferromagnetic resonance), and structural factors such as porosity and grain size. The Co^2+^/Al^3+^ co-substitution adjusts these parameters simultaneously by modifying lattice strain, anisotropy, and carrier mobility. As a result, the intermediate substituted samples (particularly *x* = 0.50) exhibit balanced impedance matching, high attenuation constant, and minimal reflected power traits ideal for electromagnetic interference shielding and stealth applications.

## 4. Conclusions

Co–Al co-substituted Ba_0.5_Sr_0.5_Y_1.0_Fe_11_O_19_ hexaferrites were successfully synthesized using a sol–gel auto-combustion method, enabling systematic control over lattice contraction, microstrain, and grain consolidation while maintaining single-phase M-type structure. The incorporation of Co^2+^ and Al^3+^ ions modified the Fe–O–Fe superexchange network and increased microstrain, contributing to improved densification and structural uniformity. FESEM, EDX, and elemental mapping confirmed homogeneous cation distribution and chemical purity across the series.

Magnetic studies revealed progressive softening of ferrimagnetic behavior due to the reduction in magnetocrystalline anisotropy and weakening of Fe^3+^–O^2−^–Fe^3+^ superexchange interactions. This enhanced domain-wall mobility, spin relaxation, and ferromagnetic resonance enabled stronger high-frequency magnetic loss. Concurrently, the dielectric response, dominated by Maxwell–Wagner interfacial polarization and suppressed hopping conduction, produced a balanced *ε*′ − *ε*″ and *µ*′ − *µ*″ behavior. These coordinated magnetic and dielectric adjustments generated near-ideal impedance matching at intermediate substitution levels, allowing efficient microwave penetration and energy dissipation.

As a result of these synergistic structural and electromagnetic modifications, the microwave attenuation performance was significantly enhanced. The optimally substituted composition (*x* = 0.50) achieved a deep reflection loss of RL_min_ ≈ −47.6 dB at 9.27 GHz (6 mm) together with an effective absorption bandwidth (EAB) of 0.61 GHz, demonstrating nearly complete microwave absorption in the X-band. The improved attenuation constant, reduced eddy-current losses, and favorable absorption-to-reflection ratio further confirm the superior energy dissipation capability of the optimized sample.

In summary, Co–Al co-doping provides an effective route for simultaneously tuning anisotropy, polarization relaxation, and impedance matching in Ba–Sr–Y hexaferrites. The resulting materials exhibit strong absorption, broadened bandwidth, and stable performance, underscoring their suitability for lightweight, high-efficiency microwave absorption and EMI-suppression applications.

## Author contributions

Pallavi S. Salunke: formal analysis, methodology, writing – original draft preparation. Manisha R. Pati: formal analysis, writing – original draft preparation. Kanak S. Alone: methodology, visualization. Akash V. Fulari: methodology, writing – original draft reparation. Maheshkumar L. Mane: methodology, writing – original draft preparation. R. H. Kadam: formal analysis, writing – reviewing and editing. Suresh T. Alone: formal analysis, methodology, writing – original draft preparation. Sagar E. Shirsath: formal analysis, writing – reviewing and editing. Vinod N. Dhage: supervision, writing – reviewing and editing.

## Conflicts of interest

Authors declared no conflict of interest.

## Data Availability

All data will be made available on reasonable request.
